# Can Hyperperfusion of Nonaerated Lung Explain COVID-19 Hypoxia?

**DOI:** 10.21203/rs.3.rs-32949/v1

**Published:** 2020-06-01

**Authors:** Jacob Herrmann, Vitor Mori, Jason H.T. Bates, Béla Suki

**Affiliations:** 1 Dept. of Biomedical Engineering, Boston University, Boston, MA, USA; 2 Dept. of Medicine, University of Vermont, Burlington, VT, USA

## Abstract

Early stages of the novel coronavirus disease (COVID-19) have been associated with ‘silent hypoxia’ and poor oxygenation despite relatively small fractions of afflicted lung. Although it has been speculated that such paradoxical findings may be explained by impairment of hypoxic pulmonary vasoconstriction in infected lungs regions, no studies have confirmed this hypothesis nor determined whether such extreme degrees of perfusion redistribution are physiologically plausible. Here, we present a mathematical model which provides evidence that the extreme amount of pulmonary shunt observed in patients with early COVID-19 is not plausible without hyperperfusion of the relatively small fraction of injured lung, with three-fold increases in regional perfusion to afflicted regions. Although underlying perfusion heterogeneity (e.g., due to gravity or pulmonary emboli) exacerbated existing shunt in the model, the reported severity of hypoxia in early COVID-19 patients could not be replicated without considerable reduction of vascular resistance in nonoxygenated regions.

## Introduction

Gattinoni et al. described disease due to the novel coronavirus (COVID-19) as following two stages, or phenotypes ^1^. Patients initially present with a “Type L” phenotype characterized by “Low” lung stiffness (normal compliance averaging 50 mL cmH_2_O^−1^) despite poor oxygenation. Type L may transition into a frequently fatal “Type H” phenotype characterized by “High” lung stiffness (reduced compliance) accompanied by features reminiscent of conventional acute respiratory distress syndrome (ARDS) ^1^. Although a strict dichotomy of phenotypes based on respiratory compliance is controversial ^2^, there are nonetheless frequent reports of severe hypoxia in patients with only a small fraction of nonaerated lung ^3-5^, including so-called ‘silent hypoxia’. Thus the early stages of COVID-19 appear to be unique and poorly understood, manifesting in the lung as peripheral lesions characterized by ground-glass opacification on computed tomography (CT) ^6,7^. Curiously, the fraction of lung affected in this way is often surprisingly low given the severity of the associated hypoxia and estimated shunt fractions (average 50%) ^8^. If one assumes that ground-glass opacification represents lung that is nonventilated, these CT studies imply abnormally high ratios of shunt fraction to nonaerated lung fraction of 3.0 for COVID-19 compared to 1.3 for ARDS ^8^.

A possible interpretation of the Type L phenotype is that a disproportionately large fraction of the pulmonary circulation is being directed through nonaerated lung ^1,5,8^. Accordingly, Gattinoni et al. hypothesized that their seemingly paradoxical observations of hypoxia and high compliance were related to an underlying impairment of hypoxic pulmonary vasoconstriction (HPV) ^8^. Normally, HPV is a feedback mechanism whereby pulmonary arterioles constrict in lung regions with poor oxygenation ^9^. This response results in increased regional vascular resistance, reduced regional perfusion, and thereby reduced overall shunt fraction. It seems reasonable, therefore, that an impairment in HPV might upset the balance between ventilation and perfusion enough to explain the intriguing clinical findings that have been reported in COVID-19 patients.

Whether the above explanation is actually plausible, however, requires a quantitative analysis of the factors responsible for determining shunt fraction, ventilation maldistribution, and their relationship to each other. For example, given a certain fraction of injured lung (F_inj_) with impaired oxygen transport, what increase in regional blood flow and hence vasodilation would be necessary to manifest a ratio of shunt fraction (F_shu_) to F_inj_ above 3? What are the limitations on oxygen diffusion in injured lung that would be compatible with clinical findings? What role might gravitational gradients play? In the present study we used a mathematical model of perfusion and oxygen transport to address these questions with the goal of determining if the altered HPV hypothesis can potentially explain the Type L phenotype of COVID-19, or whether we need to look for an alternative explanation.

## Results

**Figure 1.**
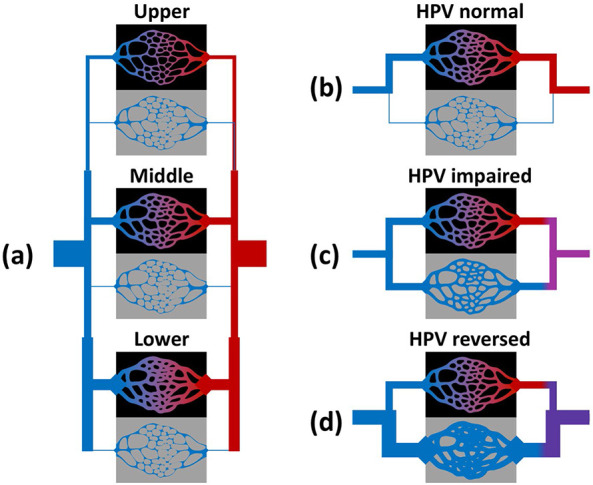
Model overview. (a) Schematic of the 6-compartment model used to simulate distributed perfusion in aerated and injured compartments at different height levels. Deoxygenated mixed venous blood (blue) passes through aerated (black) or injured (grey) compartments, and returns to the oxygenated mixed arterial blood (red). Vascular resistance in each compartment was determined by height level as well as the degree of oxygenation or injury. Hypoxic pulmonary vasoconstriction (HPV) could be either (b) “normal” with reduced perfusion to regions of low end-capillary oxygen content, (d) “impaired” with no response, or (d) “reversed” with increased perfusion to injured regions.

The lung model was partitioned into 6 compartments (Figure 1), representing 1 injured and 1 normal compartment at each of 3 height levels. Each compartment was perfused, receiving deoxygenated mixed venous blood and returning end-capillary blood with oxygen content determined by injury severity. Perfusion distribution in the model reflected the relative vascular resistance in each compartment. Baseline resistances were determined by a specified baseline perfusion gradient, defined as half the range of perfusion across all height levels divided by the average. Vascular resistance was then adjusted in injured regions to reflect possible abnormalities arising in COVID-19. Three types of modification were examined: 1) normal HPV function increased resistance exponentially in regions with low P_c_O_2_; 2) impaired HPV function produced no change in resistance; and 3) “reversed” HPV reduced resistance regardless of oxygenation. Measured outcomes included F_shu_, ratio F_shu_:F_inj_, and ratio P_a_O_2_:F_i_O_2_.

**Figure 2.**
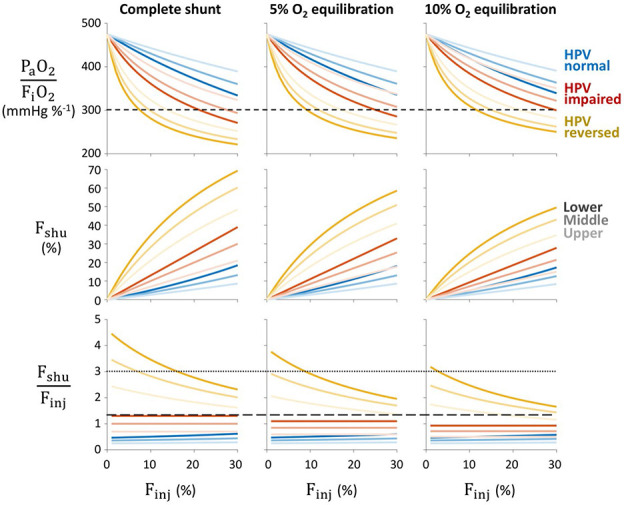
Effects of alterations to hypoxic pulmonary vasoconstriction (HPV). Severity of pulmonary shunt with respect to fractional injury extent (F_inj_), type of HPV modification (color), injury location within one height level (light to dark). Rows correspond to the ratio of arterial oxygen tension to inspired oxygen fraction (P_a_O_2_:F_i_O_2_), shunt fraction (F_shu_), and ratio of shunt fraction to injured fraction (F_shu_:F_inj_). Columns correspond to varying degrees of impaired oxygen equilibration between capillary blood and alveolar gas in the injured region. Baseline perfusion gradient was 30%, and reversed HPV was modeled with 72% reduction of vascular resistance in injured regions. Short-dashed line in the top row indicates P_a_O_2_:F_i_O_2_ = 300 mmHg %^−1^, a threshold for ARDS. Dotted and long-dashed lines in the bottom row indicate F_shu_:F_inj_ ratios of 3.0 and 1.3, respectively, the values reported by Gattinoni et al. for COVID-19 and ARDS patients, respectively ^8^.

The variability in F_shu_:F_inj_ with respect to injury location and HPV alterations is shown in Figure 2. For simplicity, the extent of injury in each simulation was restricted to only 1 height zone: lower, middle, or upper. Normal HPV function results in the lowest pulmonary shunt fractions and lowest degree of hypoxia, preventing P_a_O_2_:F_i_O_2_ < 300 mmHg %^−1^ until F_inj_ > 30%. Given a relatively small fraction of injured lung, with F_inj_ ranging from 0 to 30%, both a complete shunt (i.e., zero oxygen uptake) and “reversal” of HPV (i.e., vasodilation in injured regions) are necessary conditions for F_shu_:F_inj_ > 2 and P_a_O_2_:F_i_O_2_ < 300 mmHg %^−1^. By contrast, impairment of HPV alone is not sufficient to produce the same level of severe hypoxia at low values of F_inj_ as found by Gattinoni et al. ^8^. With HPV impairment, F_shu_ more closely follows F_inj_ such that the ratio of F_shu_ to F_inj_ lies between 0.7 and 1.3. For all considered alterations to HPV, focusing the injury in the lower zone (i.e., those with higher baseline perfusion) results in higher F_shu_ and worse hypoxia. Interestingly, as F_inj_ decreases in the reversed HPV model, the F_shu_:F_inj_ ratio increases, indicating that F_shu_ decreases more slowly than F_inj_. Note that the impaired HPV model represents unaltered vascular resistances from baseline values, and therefore corresponds to a model with relatively uniform perfusion distribution.

**Figure 3.**
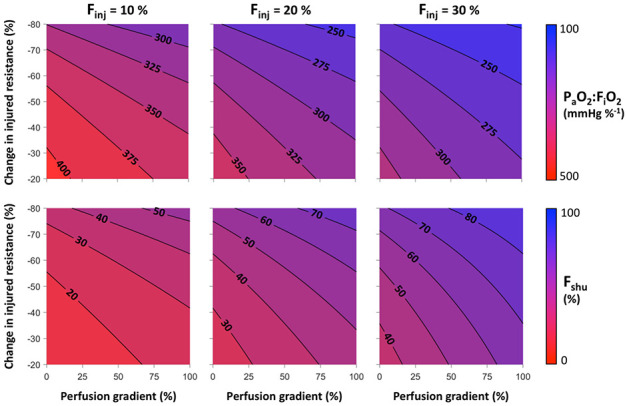
Hypoxia severity maps with respect to perfusion gradient and percent change in vascular resistance within the injured compartment of the lower lung zone. Top row shows contours of the ratio of arterial oxygen tension to inspired oxygen fraction (P_a_O_2_:F_i_O_2_). Bottom row shows contours of the shunt fraction (F_shu_). Columns represent different levels of the fraction of lung injured (F_inj_).

The interplay between baseline perfusion gradients and vasodilation in the HPV reversal model is shown in Figure 3. Baseline perfusion gradient varied between 0 and 100% representing a range of perfusion heterogeneity from uniform with ⅓-⅓-⅓ distribution at 0% gradient to 0-⅓-⅔ distribution at 100% gradient. Pulmonary shunt and hypoxia both become more severe with increases in either the vasodilation of injured regions or the baseline perfusion gradient. Both of these factors determine the overall degree of perfusion heterogeneity in the injured lung, and in the specific case of injury focused in the lower lung, both contribute to enhanced perfusion to the injured region. Hypoxia and shunt are more sensitive to the degree of vasodilation compared to the baseline gradient. The ratio of F_shu_:F_inj_ = 3 is represented in each panel by contours of F_shu_ at 30, 60, and 90% for F_inj_ at 10, 20, and 30%, respectively. Note that baseline perfusion gradient does not explicitly require the definition of upright vs. supine vs. prone positioning, but instead simply reflects discrepant perfusion in 3 arbitrary lung compartments.

## Discussion

Our analysis based on a simple mathematical model of perfusion in normal and shunted compartments suggests that the extreme amount of pulmonary shunt observed in patients with early stage severe COVID-19 is not plausible without hyperperfusion of the relatively small fraction of injured lung, with up to 3-fold increases in regional perfusion to the afflicted regions. Furthermore, the obstruction to oxygen diffusion in the injured regions must be nearly complete such that there is less than 5% equilibration between alveolar gas and end-capillary blood.

The Type L early stage of COVID-19 described by Gattinoni et al. ^1,8^, characterized by severe hypoxia but relatively normal lung compliance, cannot be recapitulated in this model without dramatic reductions to vascular resistance in the injured regions. To replicate the reported values for F_shu_ of 50% and F_shu_:F_inj_ of 3 (implying F_inj_ of 17%), the model requires reductions in injured resistance of 60 to 70%, depending on the baseline perfusion gradient (Figure 3). Approximating vascular resistance using the Hagen-Poiseuille equation, this change in resistance corresponds to an increase in vascular diameter of 26 to 35%. Whether this degree of vasodilation is physiologically plausible is uncertain. Vasodilation using inhaled nitric oxide has been reported to decrease total pulmonary vascular resistance by up to 50% ^10,11^. If the model-predicted 60 to 70% reduction of resistance is possible, it likely represents maximal vasodilation and recruitment of pulmonary capillaries. Although speculative, it may be possible that COVID-19 interferes with the HPV feedback mechanism in such a way that pulmonary arterioles do not constrict, and potentially dilate, in injured lung regions in which there is little or no oxygen transport into the blood. The virus is known to enter cells via the ACE2 receptor, and may potentially interfere with the renin-angiotensin system in ways that alter pulmonary vascular tone ^12^. Although it is reported that later stages of the disease are characterized by downregulation of ACE2 and vasoconstriction (promoting ARDS) ^12-14^, it is possible that earlier stages instead promote local vasodilation or impairment of HPV ^5,15^. Other evidence of vasodilation due to COVID-19 includes recent discovery of cardiovascular complications reminiscent of vasodilatory shock and Kawasaki disease ^16^, which is associated with weakened walls of the coronary artery.

Impairment of HPV alone cannot reproduce the same extreme values of F_shu_:F_inj_ > 2 in our model. Instead, the F_shu_:F_inj_ ratio in the impaired HPV model is limited by the magnitude of the baseline perfusion gradient. With zero baseline perfusion gradient and impaired HPV, vascular resistance is uniform across all compartments, and thus the fraction of shunted blood flow is equal to the fraction of lung with impaired oxygen transport (F_shu_:F_inj_ = 1). Heterogeneous perfusion may increase the risk for larger F_shu_, especially when the injured region also receives more baseline perfusion (see Figure 2). The value of F_shu_:F_inj_ = 1.3 for typical ARDS reported by Gattinoni et al. ^8^ is well-matched in our model with a moderate baseline perfusion gradient of 30%, impairment of HPV, and injury focused in the lower compartment (see Figure 2). This suggests that HPV impairment (e.g., due to sedatives or anesthetic agents with vasodilating effects) and prevalence of derecruitment in the gravitationally dependent lung (typically dorsal regions in a supine patient) are plausible factors contributing to the observed F_shu_:F_inj_ ratio of 1.3.

In COVID-19, the lower left and lower right lobes are most commonly afflicted according to radiographic abnormalities ^7,17^, and these are typically the gravitationally dependent regions of the lung in either upright or supine positioning. However even with an extreme baseline perfusion gradient of 100% (corresponding to 0-⅓-⅔ distribution), F_shu_:F_inj_ in the model is still limited to 2 at most. For example, in Figure 3, F_shu_:F_inj_ does not exceed 3 even at 100% baseline perfusion gradient until the resistance reduction is 40% for F_inj_ = 10%, or 55% for F_inj_ = 20%. Therefore it appears unlikely that the degree of pulmonary shunt reported in COVID-19 patients (F_shu_ = 50% and F_shu_:F_inj_ = 3) could occur without a substantial degree of vasodilation and hyperperfusion in the small fraction of injured lung, even when considering the possibility that one-third of the lung is physiologic dead space.

It should be noted that the model assumptions do not require gravity to explain the presence of a baseline perfusion gradient. Given arbitrarily defined compartments, differences in regional perfusion could also represent conditions manifesting abnormal perfusion defects such as pulmonary emboli. Coagulation and thrombosis have been identified as symptoms of COVID-19, and in many cases are associated with mortality due to stroke, myocardial infarction, or pulmonary embolism ^13,18,19^. Pulmonary embolism may reduce or eliminate perfusion to well-aerated or ventilated lung regions, resulting in physiologic dead space and redistribution of perfusion to other lung regions. This alone does not necessarily produce hypoxia, but can exacerbate hypoxia if perfusion is redistributed to regions of pulmonary shunt. In our simple model, a baseline perfusion gradient of 100% corresponds to zero perfusion in one-third of the lung, which may be interpreted similarly to the result of severe thrombotic pulmonary emboli. Even in this case, F_shu_:F_inj_ is still limited to at most 2 with only impaired HPV, and vasodilation in injured regions remains necessary to explain F_shu_:F_inj_ ≥ 3. A case report using dual-energy CT demonstrated no indications of pulmonary emboli in the well-aerated lung, but rather vasodilation of pulmonary arteries and hyperperfusion adjacent to infected regions ^5^.

Compounding the effects of large perfusion defects with “reversed” HPV, a given ratio of F_shu_:F_inj_ may be obtained at a lower level of vasodilation in the injured region (see Figure 3). A recent study of COVID-19 patients requiring mechanical ventilation reported physiologic dead space fractions as high as 45% at the time of intubation, as well as moderate reductions in compliance ^20^. Although this cohort may represent a later stage of COVID-19, these findings support the notion that disease progression is accompanied by perfusion redistribution away from aerated regions. If thrombotic pulmonary emboli occur during the early stages of COVID-19 as well, this could amplify the apparent F_shu_ and hypoxia. In cases of increased physiologic dead space, ratios of F_shu_:F_inj_ = 3 may occur with lower, more plausible reductions of resistance in injured regions (30 to 50%), compared to the 60 to 70% reduction required without considering any physiologic dead space. Another factor that may contribute to systemic hypoxia is increased oxygen uptake by lung tissues, which may account for up to 20% of total oxygen metabolism in patients with lung injury compared to only 5% at baseline ^21^.

Reports of “silent hypoxia” in early stages of COVID-19 ^3^ may reflect the Type L phenotype. It is intriguing that hypoxia in these patients is not associated with hypercapnia. One might assume that severe pulmonary shunt would produce deficiencies in both oxygen and carbon dioxide exchange. Hypercapnia is less likely to be “silent”, considering that even small increases in arterial carbon dioxide tension elicit feedback response from pH-sensitive central chemoreceptors to increase respiratory drive. Although our model suggests that oxygen equilibration must be less than 5% to produce high ratios of F_shu_:F_inj_, it is possible that elimination of carbon dioxide is less impaired given that diffusion of carbon dioxide across the alveolar-capillary membrane is roughly 20-fold faster than that of oxygen ^3^.

Pulmonary vasodilators such as inhaled nitric oxide, sildenafil, and angiotensin-(1,7) are currently involved in clinical trials for treatment of COVID-19 (e.g., ClinicalTrials.gov identifiers NCT04290871, NCT04304313, NCT04332666). Notwithstanding other systemic effects of pharmacological interventions, our model suggests that vasodilation throughout the noninjured lung may counterbalance disease-induced vasodilation in the injured regions, restoring a more uniform baseline perfusion distribution similar to the impaired HPV model. Vasodilation may also reduce the severity of thrombotic pulmonary emboli, thereby reducing physiologic dead space and perfusion heterogeneity. Prone positioning is another intervention which may reduce baseline perfusion gradients compared to supine positioning ^22^, and is also reportedly beneficial for COVID-19 patients ^23,24^. Therefore, our model may explain a mechanism by which pulmonary vasodilators and prone positioning may improve ventilation-to-perfusion matching and reduce hypoxia, perhaps providing palliative care for COVID-19 patients.

It should be noted that no consensus has yet been established for these interventions, current evidence is largely anecdotal, and the theories proposed herein based on our simple model are speculative. The direct implications of this study are furthermore limited to palliative care, and cannot be applied to identify or verify underlying viral mechanisms that initiate or progress the disease, or to explain why asymptomatic patients exhibit radiographic indicators of viral pneumonia ^7^. It should also be noted that our model was designed to represent only early stages of severe COVID-19 (i.e., before it develops into full-blown ARDS) where there is only a small fraction of injured lung and before the initiation of mechanical ventilation or other respiratory support. The purpose of the model was to quantitatively assess the plausibility of the hypothesis that severe hypoxia in early COVID-19 is the result of hyperperfusion within a small amount of injured lung. The model demonstrates that vasodilation of injured, unoxygenated regions appears to be a necessary feature of early COVID-19 to explain the reported severity of hypoxia, with or without shunt amplification by thrombotic pulmonary emboli.

## Methods

The lung model was partitioned into 6 compartments (Figure 1), representing 1 injured and 1 normal compartment at each of 3 height levels with different gravitational potentials corresponding to West zones ^25^. Each compartment was perfused, receiving deoxygenated mixed venous blood and returning end-capillary blood with oxygen content determined by injury severity. The model described time-averaged gas exchange, i.e., neglecting within-breath and within-beat fluctuations. Normal lung compartments had normal oxygen diffusion such that end-capillary oxygen tension (P_c_O_2_) equilibrated with alveolar oxygen tension (P_A_O_2_). Injured lung compartments had limited or zero oxygen diffusion such that P_c_O_2_ was either equal to mixed venous oxygen tension (P_v_O_2_) or a weighted average of P_v_O_2_ and P_A_O_2_:

$$P_{c}O_{2}=P_{v}O_{2}+B\cdot (P_{A}O_{2}-P_{v}O_{2})$$

Note that in the normal lung compartments, B was assumed to have a value of 1. A value of 0 for B corresponds to a complete shunt with no oxygen diffusion. Oxygen tensions were assumed to be P_v_O_2_ = 40 mmHg and P_A_O_2_ = 100 mmHg, representing patients upon admission without supplemental oxygen. These values result from the alveolar gas equation, with 21% inspired oxygen, 47 mmHg water vapor pressure at 37 C, 40 mmHg arterial carbon dioxide tension, and 0.8 respiratory quotient:

$$P_{A}O_{2}=0.21\cdot (760\;\mathrm{mmHg}-47\;\mathrm{mmHg}-\frac{40\;\mathrm{mmHg}}{0.8}$$

Perfusion distribution in the model reflected the relative vascular resistance in each compartment. First, baseline resistances (R_bas_) were determined to establish a baseline perfusion gradient in 3 equal-sized normal compartments (i.e., in the absence of any injury). Baseline perfusion gradient was defined as half the range of perfusion across all height levels divided by the average. Baseline vascular resistance (R_bas_) at each height level (h), relative to baseline total pulmonary vascular resistance (PVR_bas_), was determined as follows:

$$R_{\mathrm{bas}}(h)=\frac{1}{3}{\mathrm{PVR}}_{\mathrm{bas}}\frac{Q_{\mathrm{tot}}}{Q_{\mathrm{bas}}(h)}$$

where Q_tot_ is total pulmonary perfusion (i.e., cardiac output), and Q_bas_ is baseline perfusion at each height level. Vascular resistance was then adjusted in injured regions to reflect possible abnormalities arising in COVID-19. Three types of modification were examined: 1) normal HPV function increased resistance exponentially in regions with low P_c_O_2_; 2) impaired HPV function produced no change in resistance; and 3) “reversed” HPV reduced resistance by a factor 0 < K < 1 regardless of oxygenation. The following equations were used:

Rinj(h)Rbas(h)={1+100e−PcO2∕10HPV normal1HPV impairedKHPV reversed}

where R_inj_ is resistance of the injured compartment. Note that resistances were defined in a volumetric manner, such that the effective compartmental resistance was inversely proportional to the fraction of lung it represented. Following these HPV modifications and now accounting for an injured compartment at each height level with altered vascular resistance, total pulmonary vascular resistance (PVR) was computed by the parallel combination of compartmental resistances.

$$\frac{1}{\mathrm{PVR}}=\frac{1}{3}\sum_{h}\left[\left(\frac{F_{\mathrm{inj}}(h)}{R_{\mathrm{inj}}(h)}\right)+\left(\frac{1-F_{\mathrm{inj}}(h)}{R_{\mathrm{bas}}(h)}\right)\right]$$

Perfusion to each n’th compartment was then allocated in inverse proportion to compartmental resistance.

$$Q_{n}=\mathrm{PVR}=\left(\frac{F_{n}}{R_{n}}\right)$$

where R_n_ is the resistance of the n’th compartment and F_n_ is the fraction of the total lung represented by that compartment. End-capillary oxygen content (C_c_O_2_) was computed based on P_c_O_2_ for each compartment:

$$C_{c}O_{2}=1.34\cdot [\mathrm{Hb}]\cdot S_{c}O_{2}+0.0031\cdot P_{c}O_{2}$$

where S_c_O_2_ is oxygen saturation of hemoglobin determined by the Severinghaus equation fit to the oxygen-hemoglobin dissociation curve ^26^:

$$S_{c}O_{2}={\left(1+\frac{23400}{P_{c}{O_{2}}^{3}+150\cdot P_{c}O_{2}}\right)}^{-1}$$

Mixed venous oxygen content (C_v_O_2_) was calculated in the same manner. Mixed arterial oxygen content (C_a_O_2_) was then determined as a perfusion-weighted average of compartmental end-capillary oxygen contents.

$$C_{a}O_{2}=\sum_{n}(C_{c}O_{2}{)}_{n}\cdot \frac{Q_{n}}{Q_{\mathrm{tot}}}$$

Shunt fraction (F_shu_) was defined as the ratio of unoxygenated to total blood flow:

$$F_{\mathrm{shu}}=\frac{C_{c*}O_{2}-C_{a}O_{2}}{C_{c*}O_{2}-C_{v}O_{2}}$$

where C_c_*O_2_ represents a normal(noninjured) compartment.

Measured outcomes included F_shu_, ratio F_shu_:F_inj_, and ratio P_a_O_2_:F_i_O_2_. Simulations were conducted over a range of F_inj_ from 0 to 30%. For example, 15% of the lower lung zone injured reflects an overall F_inj_ of 5%, because the lower zone represents 1/3 of the total lung. For simplicity, the extent of injury in each simulation was restricted to only 1 height zone: lower, middle, or upper. Baseline perfusion gradient varied between 0 and 100% representing a range of perfusion heterogeneity from uniform with ⅓-⅓-⅓ distribution at 0% gradient to 0-⅓-⅔ distribution at 100% gradient. The degree of limited oxygen diffusion in the injured compartment varied between 0 and 20%, where 0% represents complete shunt and 100% represents complete equilibration with alveolar gas.

## Code Availability

A Matlab script for evaluating the mathematical model described herein is available as supplementary material.

## Supplementary Material

Supplement
